# Supplementary material for: Ketoprofen-Based Polymer-Drug Nanoparticles Provide Anti-Inflammatory Properties to HA/Collagen Hydrogels
Source: J Funct Biomater. 2023 Mar 17;14(3):160. doi: 10.3390/jfb14030160 (PMC10059015; doi:10.3390/jfb14030160)
Supplement: Supplementary file 1 [file jfb-14-00160-s001.zip › jfb-2251944-supplementary.pdf]

Journal of
Functional
Biomaterials

MDPI

Supplementary Materials: Ketoprofen-Based Polymer-Drug Nanoparticles Provide Anti-Inflammatory Properties to HA/Collagen Hydrogels

Norbert Halfter [1,†], Eva Espinosa-Cano [2,3,†], Gloria María Pontes-Quero [2,3], Rosa Ana Ramírez-Jiménez [2,3], Christiane Heinemann [1], Stephanie Möller [4], Matthias Schnabelrauch [4], Hans-Peter Wiesmann [1], Vera Hintze [1,*] and Maria Rosa Aguilar [2,3,*]

[1] Institute of Materials Science, Max Bergmann Center of Biomaterials, Technische Universität Dresden, Budapester Straße 27, 01069 Dresden, Germany
[2] Group of Biomaterials, Institute of Polymer Science and Technology ICTP-CSIC, C/Juan de la Cierva 3, 28006 Madrid, Spain
[3] CIBER de Bioingeniería, Biomateriales y Nanomedicina, Instituto de Salud Carlos III, C/Monforte de Lemos 3/5, 28029 Madrid, Spain
[4] Biomaterials Department, INNOVENT e.V, Prüssingstraße 27B, 07745 Jena, Germany
* Correspondence: vera.hintze@tu-dresden.de (V.H.); mraguilar@ictp.csic.es (M.R.A.)
† These authors contributed equally to this work.

Table S1. Used volumes for gel loading with NP for cell experiments and determination of properties. V_{NP} and $V_{acetic\ acid}$ were mixed before incubation of the gels.

Label	c HA-MAC [mg/mL] [1]	c coll [mg/mL] [1]	Gel type	V of 1 mg/mL NP per HG [µL] [2]	V of 0.1 M Acetic acid [µL]
10HA 40NP	10	0.5	HG	65	185
10HA 120NP	10	0.5	HG	195	55
CL-10HA 40NP	*10*	*0.5*	*HG*	*40[a)]*	-
30HA 40NP	30	0.5	HG	119	131
cryo 10HA 40NP	10	0.5	CG	41	209

[1] before the addition of LAP; [2] calculation was based on the first experiments of the release experiments for 1 d.

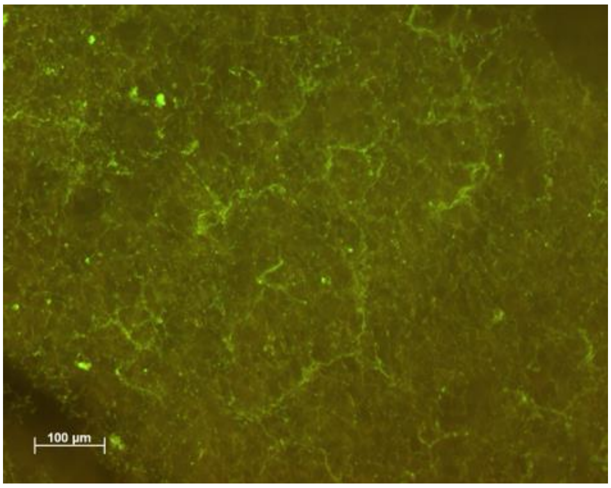

Figure S1. Fluorescence image of cryo 10HA 40NP after extraction with EtOH.

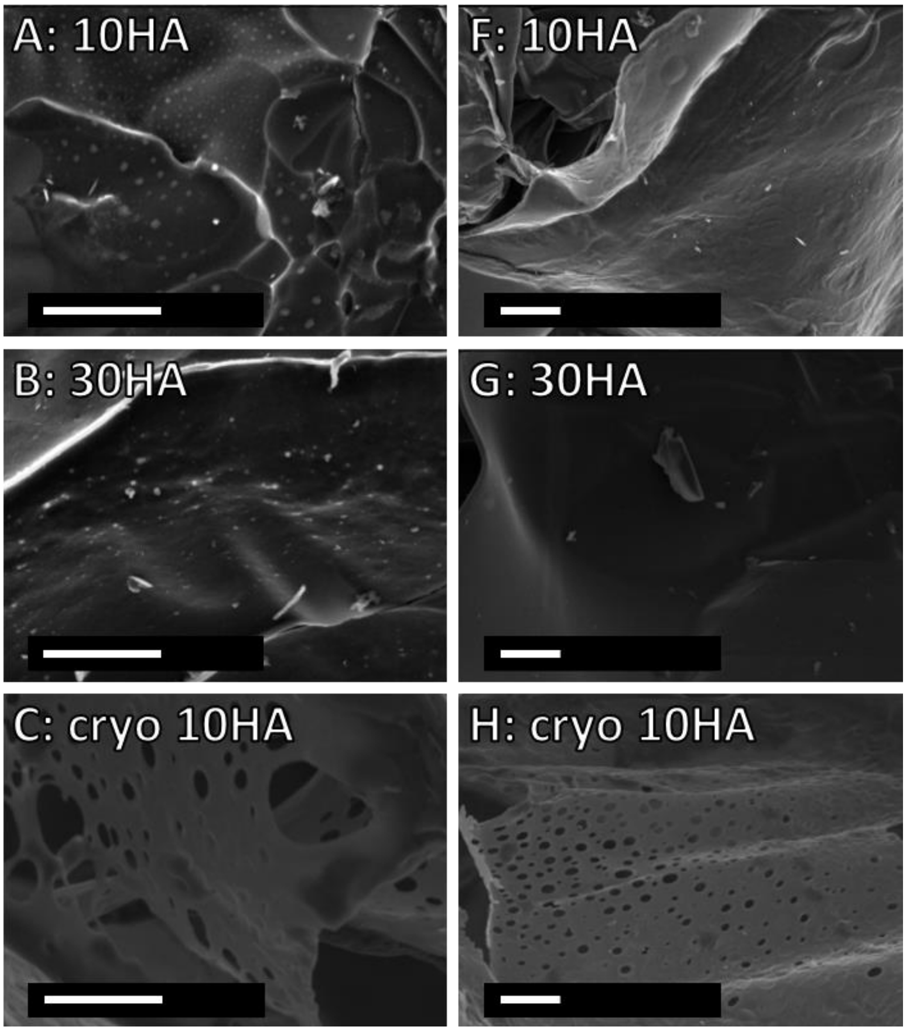

Figure S2. Top view (A-C, magnification: 40,000x) and cross-section (D-F, magnification: 20,000x) of freeze-dried gels without NP. Scale bar = 2 µm.

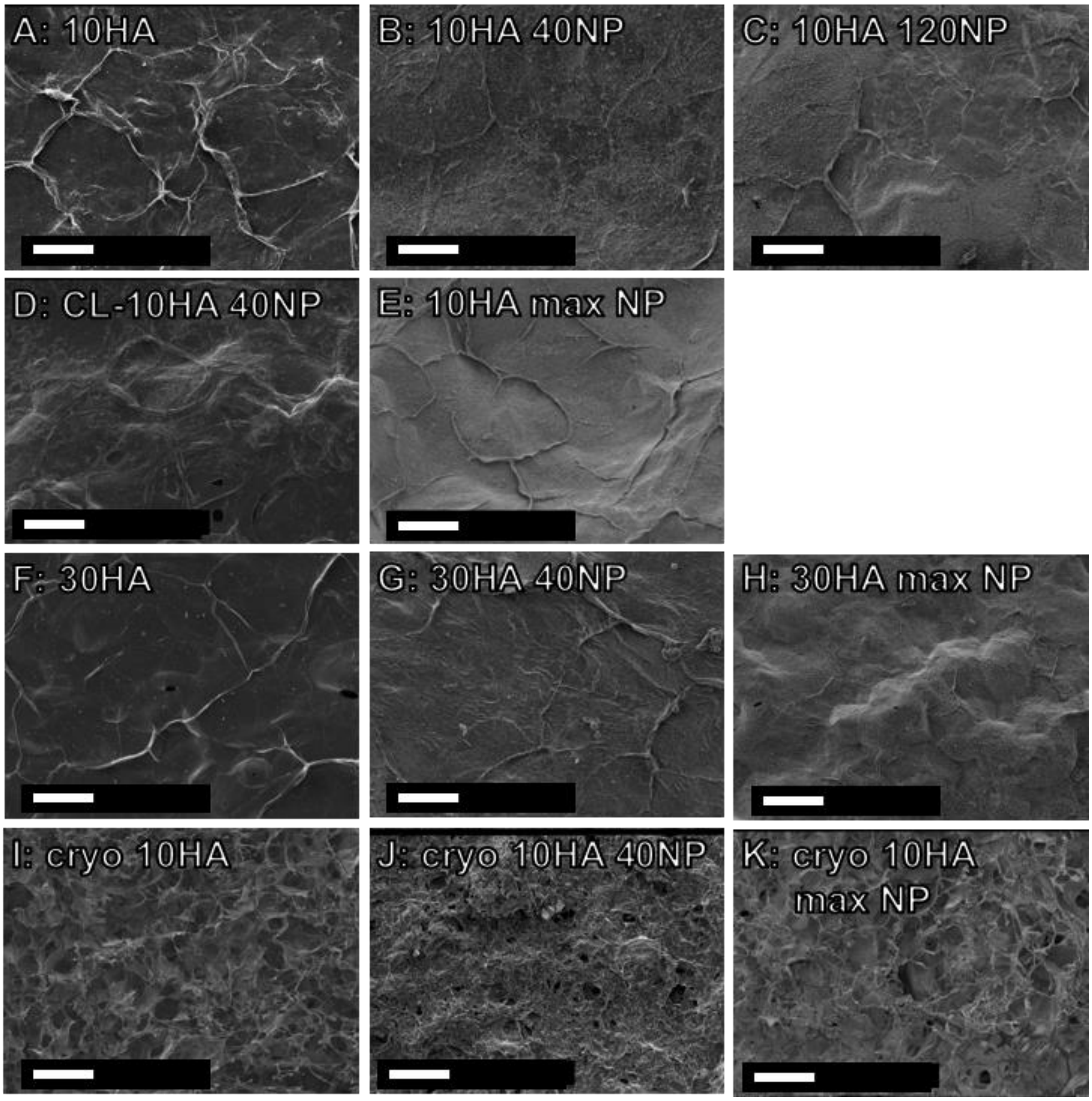

Figure S3. Top view of freeze-dried gels. Scale bar 50 μM. Magnification for all images was 1000x.

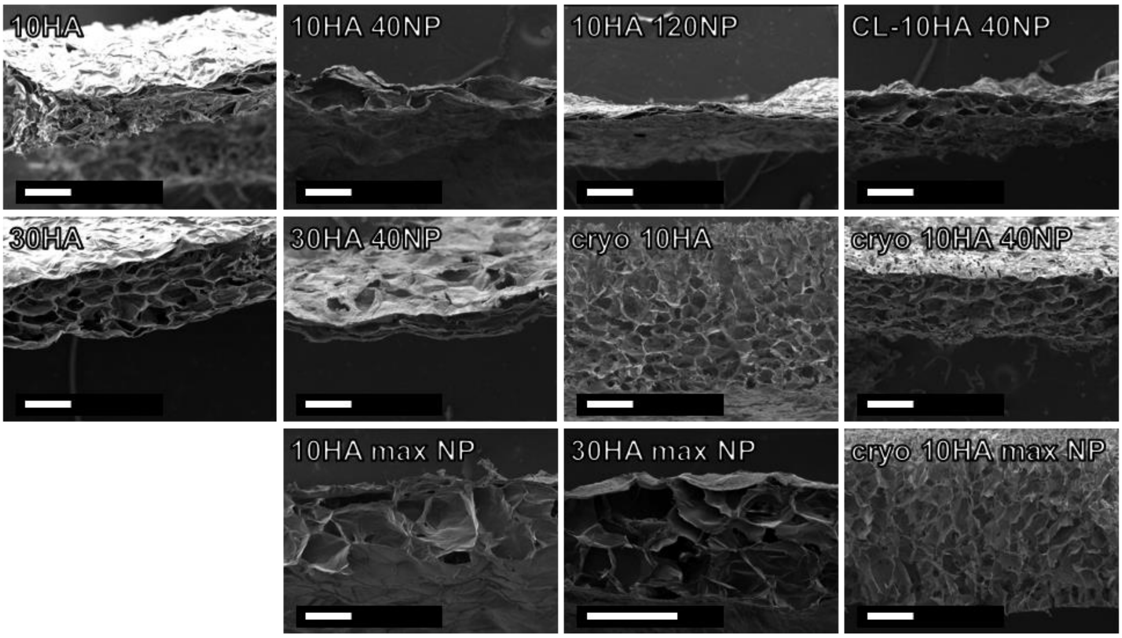

Figure S4. Cross-section of freeze-dried gels. Scale bar 100 µm (magnification: 500x, except 30HA max NP, there magnification is 1000x).

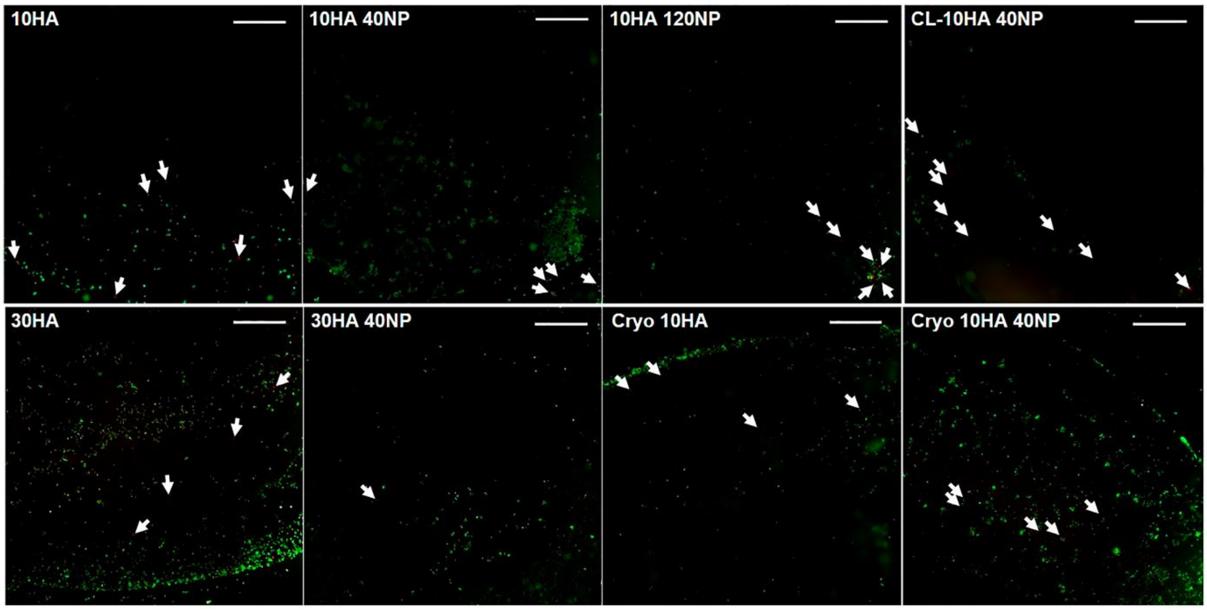

Figure S5. Cell viability. Representative fluorescent images of RAW264.7 stained with Calcein AM (living cells in green) and ethidium homodimer (dead cells in red and indicated with arrows) after 48 h using the Live/Dead™ assay. Scale: 1 mm.

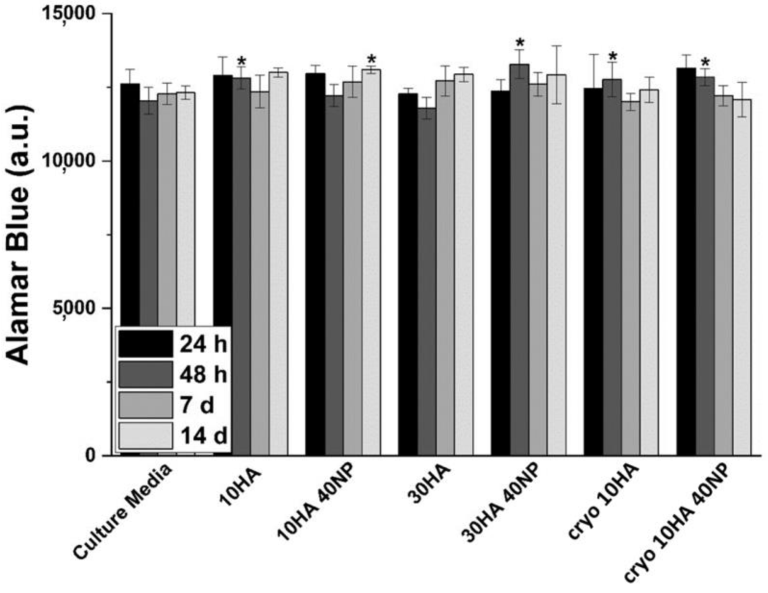

Figure S6. Effect of gel extracts on cell proliferation using an AlamarBlue® assay. Data are represented as mean ± SD values. ANOVA between cells treated with gel extracts and culture media controls was performed at each time point (*p < 0.05).

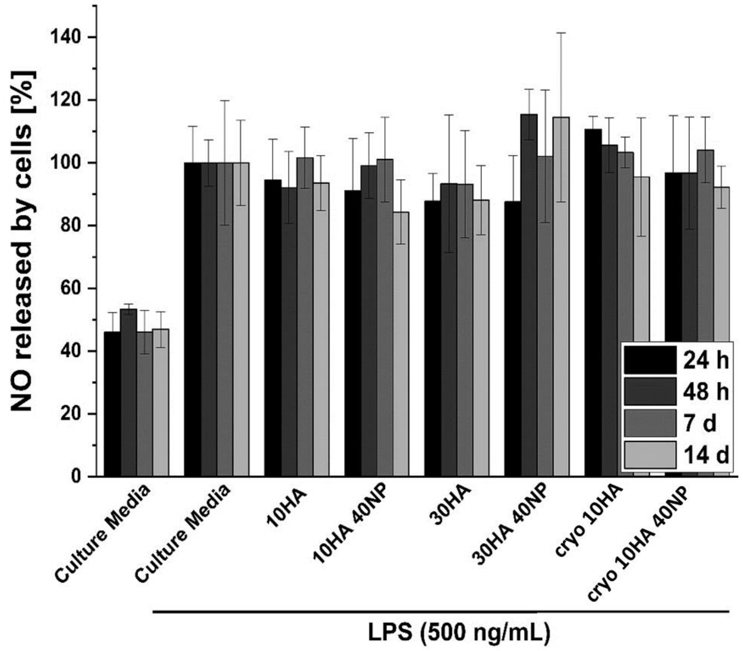

Figure S7. Effect of gel extracts on NO production in LPS-stimulated RAW264.7. Mean ± SD values are relative to control LPS-stimulated culture media cells, in which NO production was taken as 100%. ANOVA between cells treated with gel extracts and culture media controls was performed at each time point (*p < 0.05).
